# 2D-to-3D image translation of complex nanoporous volumes using generative networks

**DOI:** 10.1038/s41598-021-00080-5

**Published:** 2021-10-21

**Authors:** Timothy I. Anderson, Bolivia Vega, Jesse McKinzie, Saman A. Aryana, Anthony R. Kovscek

**Affiliations:** 1 Department of Electrical Engineering, Stanford University, Stanford, CA 94305 USA; 2 Department of Energy Resources Engineering, Stanford University, Stanford, CA 94305 USA; 3 Department of Chemical Engineering, University of Wyoming, Laramie, WY 82071 USA

**Keywords:** Carbon capture and storage, Fossil fuels, Materials for energy and catalysis

## Abstract

Image-based characterization offers a powerful approach to studying geological porous media at the nanoscale and images are critical to understanding reactive transport mechanisms in reservoirs relevant to energy and sustainability technologies such as carbon sequestration, subsurface hydrogen storage, and natural gas recovery. Nanoimaging presents a trade off, however, between higher-contrast sample-destructive and lower-contrast sample-preserving imaging modalities. Furthermore, high-contrast imaging modalities often acquire only 2D images, while 3D volumes are needed to characterize fully a source rock sample. In this work, we present deep learning image translation models to predict high-contrast focused ion beam-scanning electron microscopy (FIB-SEM) image volumes from transmission X-ray microscopy (TXM) images when only 2D paired training data is available. We introduce a regularization method for improving 3D volume generation from 2D-to-2D deep learning image models and apply this approach to translate 3D TXM volumes to FIB-SEM fidelity. We then segment a predicted FIB-SEM volume into a flow simulation domain and calculate the sample apparent permeability using a lattice Boltzmann method (LBM) technique. Results show that our image translation approach produces simulation domains suitable for flow visualization and allows for accurate characterization of petrophysical properties from non-destructive imaging data.

## Introduction

The transition to a sustainable energy future requires a combination of greenhouse gas sequestration, long-term adoption of renewable sources of energy, and near-term fuel switching to cleaner available energy resources^1^. Approaches that could contribute to these goals include CO_2_ sequestration, subsurface H_2_ or compressed air storage, and natural gas recovery^2–6^. One important requirement for scalable and sustainable implementation of these technologies is more rapid and reliable characterization of porous media transport properties in order to identify viable candidate formations.

Image-based characterization—and nanoimaging in particular—combined with digital rock physics are critical to understanding geological porous media at the pore scale^7–10^. Non-destructive imaging modalities such as transmission X-ray microscopy (TXM), scanning transmission electron microscopy (STEM), and X-ray spectroscopy (XRS) allow for characterizing the petrophysical properties of a sample while preserving it for future experimentation^11–16^. On the other hand, destructive imaging modalities such as focused-ion beam scanning electron microscopy (FIB-SEM) obtain high-contrast/high-resolution images at the expense of destroying the sample^17^.

**Figure 1:**
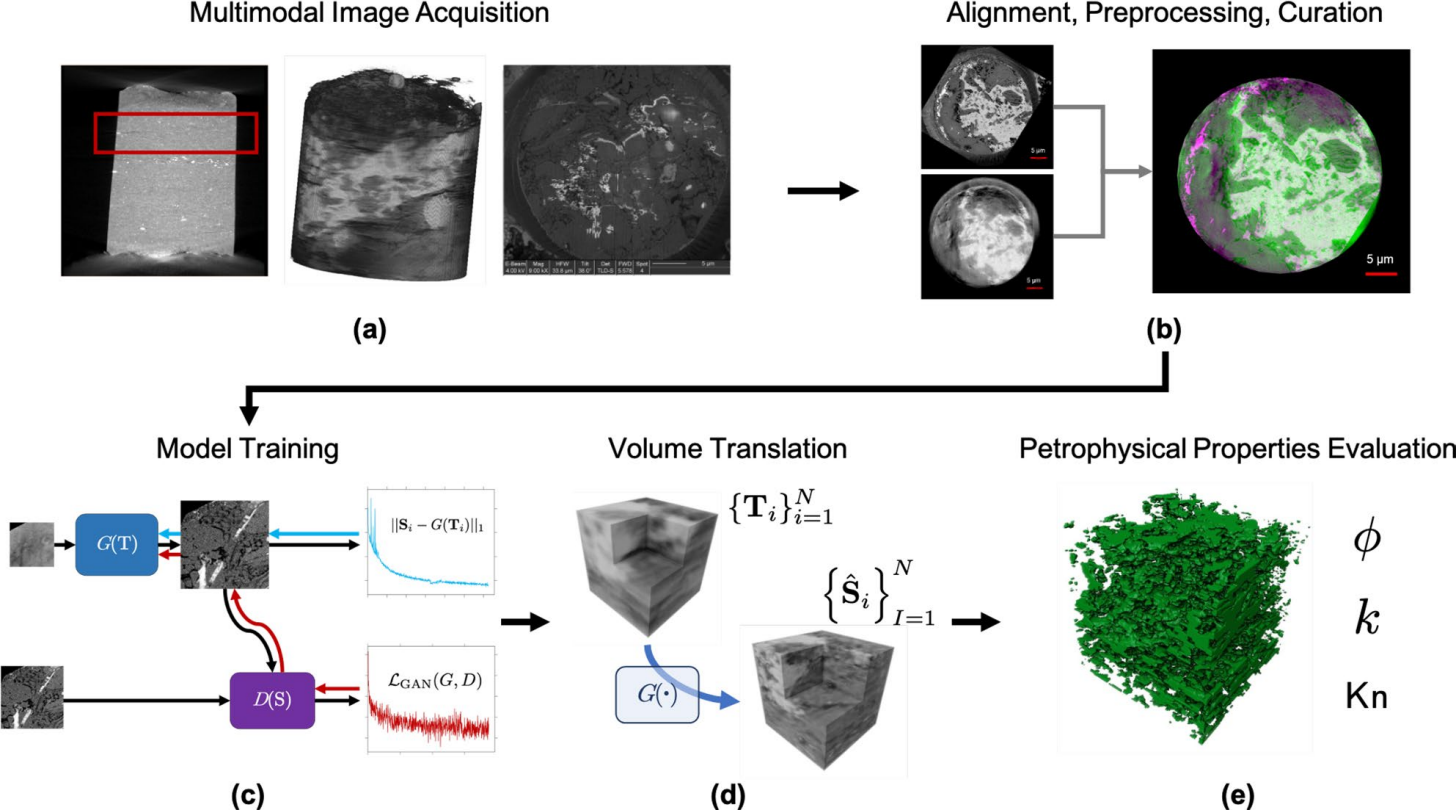
Multimodal image characterization workflow. (**a**) Multimodal image acquisition for a Vaca Muerta shale sample. After initial $\mu$-CT imaging (left), a region with minimal pyrite deposits is selected and milled to a 30 $\mu$m diameter cylindrical plug. This is imaged to produce a TXM image volume (middle). The plug is then successively focused ion beam milled and SEM imaged to create FIB-SEM image slices (right). (**b**) FIB-SEM and TXM image slices are aligned and normalized to create a paired 2D image dataset. (**c**) Image translation models are trained to predict FIB-SEM images from TXM images. These models include a continuity loss term to improve 3D volume translation when only 2D images are available. (**d**) Image volumes are predicted using the trained model. The volumes are synthesized by passing *x*–*y* image slices through the network independently to create the sequence of predicted FIB-SEM images. (**e**) Predicted FIB-SEM volumes are segmented into a simulation domain and petrophysical properties evaluated using digital rock physics techniques. Software: Powerpoint www.microsoft.com, Avizo www.thermofishwer.com.

An emerging area of image-based porous media characterization is isoscale multimodal imaging, where two or more imaging modalities at the same resolution are acquired to characterize a single sample^11,18^. Multimodal imaging and image data translation is a promising approach to characterization that enables the advantages of two or more imaging modalities, namely sample-preservation and high-resolution^19^. In a multimodal imaging workflow such as that shown in Fig. 1, a sample is imaged using two or more imaging modalities at the same resolution, a model is trained to translate between modalities, and the synthesized images used to estimate petrophysical properties of the sample^20–22^. Multimodal image prediction or enhancement is common in medical imaging^23–25^, but this methodology has yet to gain prominence in source rock characterization. Indeed, little work exists on applying deep learning models to porous media images, with most work focusing on synthesizing^26–28^ or segmenting images^29^ rather than translating images across modalities.

Image prediction using multimodal imaging involves elements of single image super-resolution (SISR) and image-to-image translation. SISR seeks to predict a high resolution image from a low resolution input. Many models have been proposed for this problem, with the most successful being dictionary methods^30–32^ and deep learning-based methods^33,34^. Image-to-image translation meanwhile seeks to translate images between domains^35^. Deep learning models for image translation include neural style transfer algorithms^36,37^, paired image translation^38^, and unpaired image translation^39^. The most common deep learning models for these tasks are based on feed-forward convolutional neural networks (CNNs) and conditional generative adversarial networks (CGANs)^38,40,41^.

3D-to-3D image volume translation typically requires paired 3D training data, and consequently is only applicable in a limited number of contexts such as medical imaging^42,43^ where sufficient amounts of aligned 3D multimodal data is available. Multimodal imaging for source rock samples often contains a mixture of 2D surface imaging modalities, such as electron microscopy, and 3D volumetric imaging, such as CT-based modalities^44^. Consequently, a persistent challenge for multimodal image-based characterization of geologic samples is predicting 3D volumes from non-destructive image data when only 2D training data is available. This problem remains relatively unexplored, and no work exists addressing volume reconstruction from microscopy data in the context of geological porous media characterization.

This work presents a method for predicting high-contrast geological porous media image volumes from low-contrast, sample-preserving input data using deep learning models trained on only 2D paired multimodal image data. We introduce a new method for regularizing training of deep learning image reconstruction models to improve 3D volume prediction from 2D training data, further develop quantitative metrics for evaluating 2D and 3D multimodal image assimilation models, and construct simulation domains from the translated volumes to evaluate flow properties of geologic samples from reconstructed image volumes. While presented in the context of geological media, the method is applicable to other materials with fine microstructure.

## Results

### Two-dimensional image reconstruction

**Figure 2:**
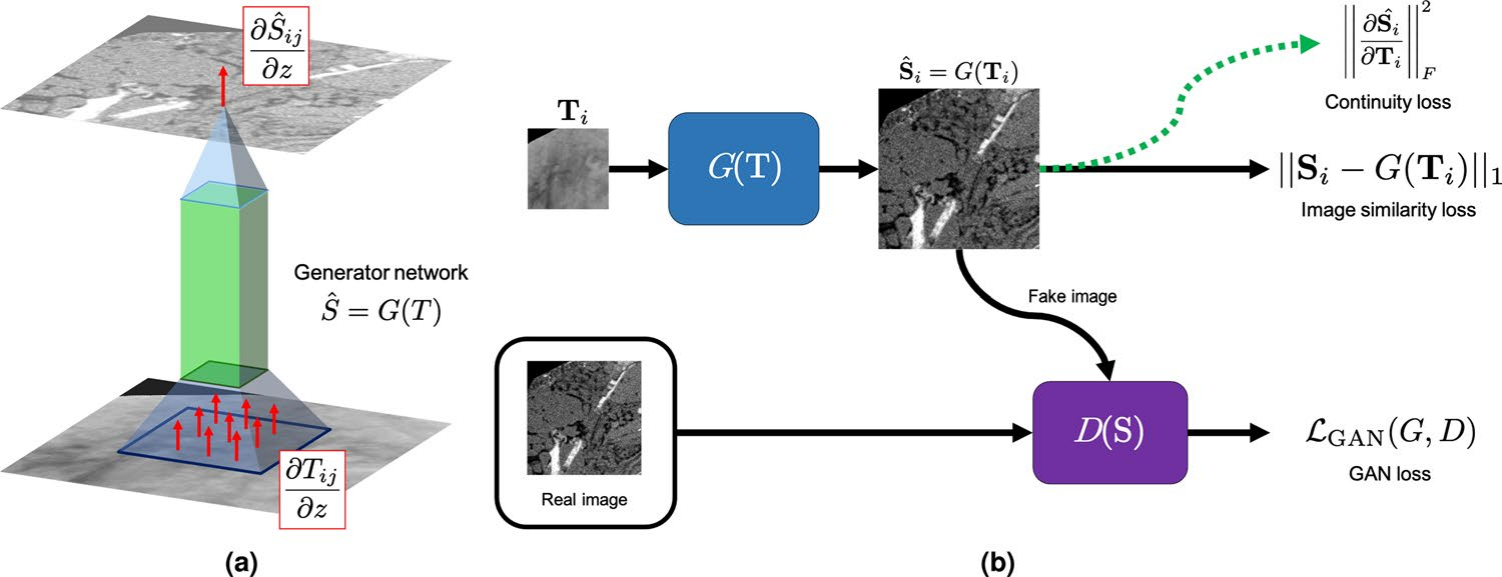
Image translation model. (**a**) Visualization of the Jacobian regularization approach proposed to improve volume prediction with 2D-to-2D models. *z*-direction gradients are assumed to be sparse and we encourage continuity in the input TXM pixels $T_{ij}$ by penalizing the training by the Jacobian of the output image with respect to the input. (**b**) SRGAN image-to-image deep learning model. The model is trained with a vanilla or Wasserstein GAN loss, $L_1$ image similarity loss, and optionally the proposed Jacobian loss to encourage continuity between input TXM slices. Software: Powerpoint www.microsoft.com.

We first examine predicting 2D FIB-SEM image patches from TXM image patches. Results for the 2D-to-2D image prediction models are summarized in Table 1 and example images shown in Fig. 2b. For unregularized models, we test several different configurations of network architectures, training objectives, and upsampling factors (for the SISR models). We also train the pix2pix with Wasserstein GAN (WGAN) loss and SRGAN 4x upsampling with vanilla GAN loss with the proposed Jacobian regularization term to improve volume generation from 2D-to-2D models (Fig. 2a). These two models were chosen because they showed the strongest performance on peak signal-to-noise ratio (PSNR), structural similarity index metric (SSIM), and structural texture similarity index metric (STSIM).

**Table 1:** Paired image similarity results.

Model	PSNR	SSIM	STSIM	Low dens.	High dens.
**Baseline Models**					
No Model	13.59	0.22	0.70	0.06	0.20
Feedforward CNN	15.82	0.20	0.72	**0**.**20**	0.18
pix2pix (Vanilla)	15.93	0.17	0.81	0.16	0.19
pix2pix (WGAN)	**16**.**57**	**0**.**25**	0.65	0.13	0.21
SRCNN 4x	15.94	0.21	0.75	0.18	0.20
SRCNN 2x	15.70	0.21	0.71	0.17	0.17
SRGAN 4x (Vanilla)	15.72	0.15	**0**.**82**	0.18	0.19
SRGAN 4x (WGAN)	16.11	0.22	0.73	0.17	0.21
SRGAN 2x (Vanilla)	16.02	0.19	0.79	0.17	0.20
SRGAN 2x (WGAN)	16.49	**0**.**25**	0.64	0.12	0.21
*z*-**regularization**					
pix2pix (WGAN)	16.19	**0**.**25**	0.61	0.12	**0**.**23**
SRGAN 4x (Vanilla)	15.94	0.17	0.80	0.16	0.20

Comparison of image reconstruction models for 2D-to-2D image translation. Metrics are calculated using 100 image patches sized $128 \times 128$ pixels sampled from held-out test set images. A greater score is better for all metrics shown. The feedforward CNN and pix2pix model use a 9-block ResNet for image translation^37^ and the SRCNN and SRGAN models use a 9-block super-resolution ResNet^34^. The pix2pix (WGAN) model performs the most strongly in terms of PSNR, SSIM, and high-density region segmentation. The SRGAN 4x (Vanilla) model performs the most strongly in terms of STSIM (perceptual similarity) and second most strongly for low-density region segmentation. The *z*-regularization slightly reduces performance for all metrics except high-density region segmentation.

**Figure 3:**
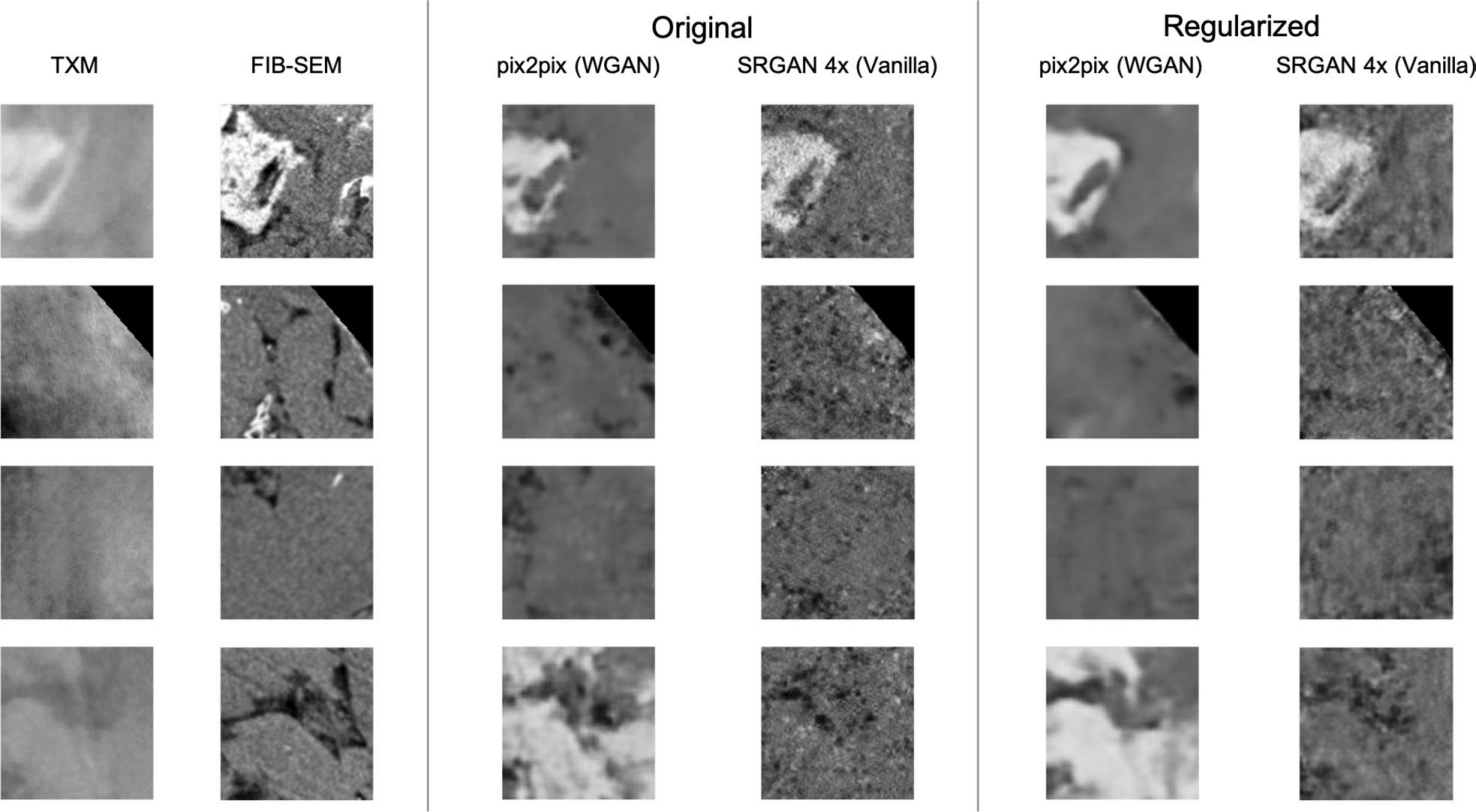
2D-to-2D image translation results. Each row contains the input TXM image patch and ground truth FIB-SEM image patch, and translated image patches for the pix2pix (WGAN) and SRGAN 4x (Vanilla) models both with and without the continuity loss term. The Jacobian loss term causes the translation models to synthesize fewer details in the images. Specifically, we observe that fewer low-density regions are synthesized by the regularized models. Software: Powerpoint www.microsoft.com.

Across all metrics, both the image translation and super-resolution models offer improvement over the unprocessed TXM images. STSIM and low density region segmentation show the largest improvement, while the high density segmentation showing minimal improvement over the raw TXM images. The WGAN models tend to outperform their vanilla GAN counterparts and GAN-based models perform a bit more strongly than the feed forward models in terms of PSNR and SSIM. The strongest performing GAN models are the WGAN models, particularly the pix2pix WGAN and SRGAN 2x WGAN models. The *z*-regularization results show that a Jacobian regularization term does not significantly impact the 2D-to-2D image translation results except for the low-density region segmentation. A comparison of image patches for original and regularized models (Fig. 3) shows that the regularization results in predicted FIB-SEM images with sparser *x* and *y* gradients and less noise. Furthermore, the *z*-regularization term causes the models to synthesize fewer low-density regions, resulting in reduced performance for low-density region segmentation.

### Three-dimensional volume prediction

**Figure 4:**
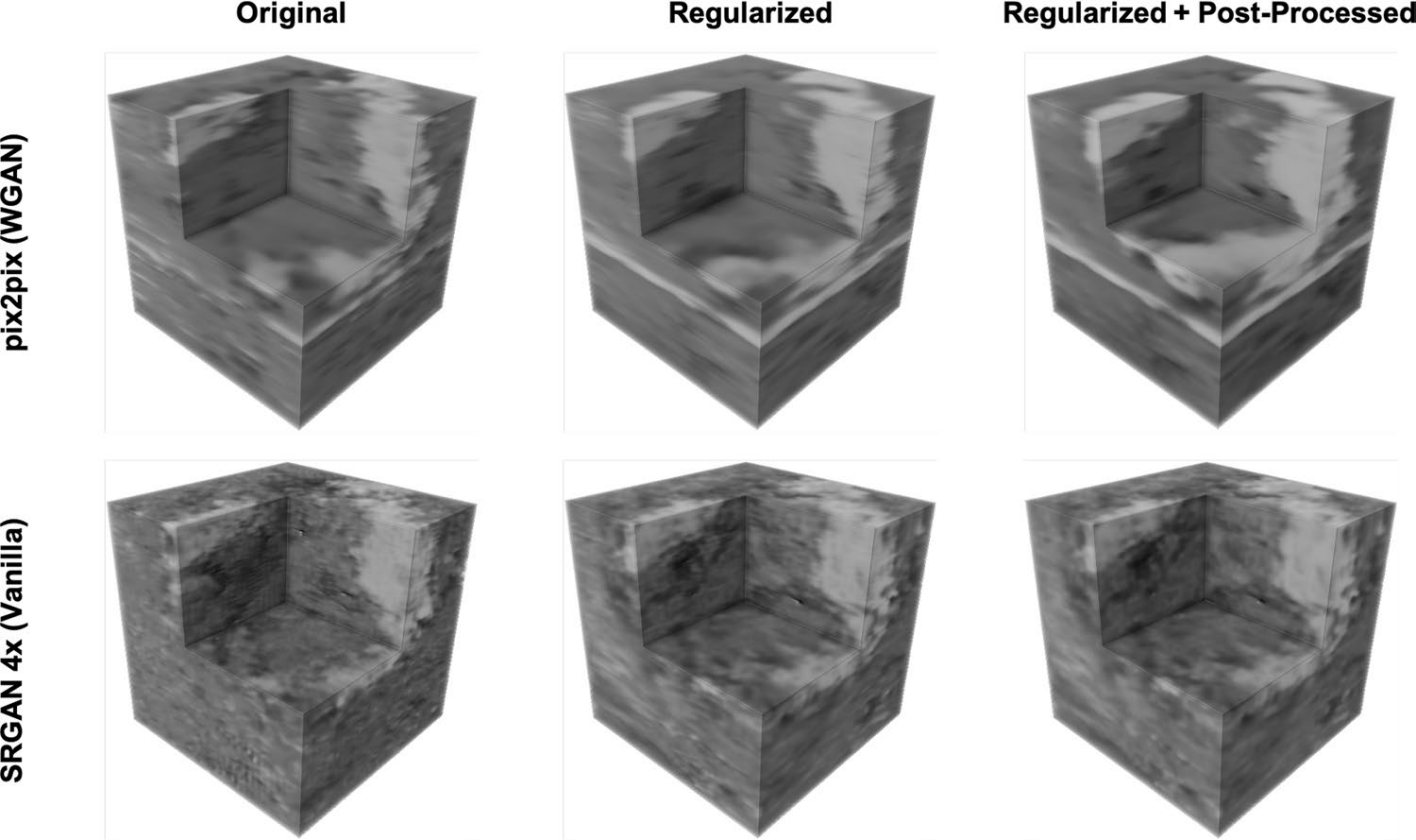
Test set volume renderings. Image volume is a $128^3$ voxel volume from a part of the imaged TXM volume held out as a test set. Volumes are evaluated for the pix2pix (WGAN) and SRGAN 4x (Vanilla) models for the original model, model trained with *z*-regularization, and regularized model with median filter post-processing using a $3 \times 1 \times 1$ structuring element. The *x*–*z* and *y*–*z* cross sections show improved *z*-direction continuity for the regularized and post-processed images. Software: Powerpoint www.microsoft.com, MATLAB www.mathworks.com.

**Figure 5:**
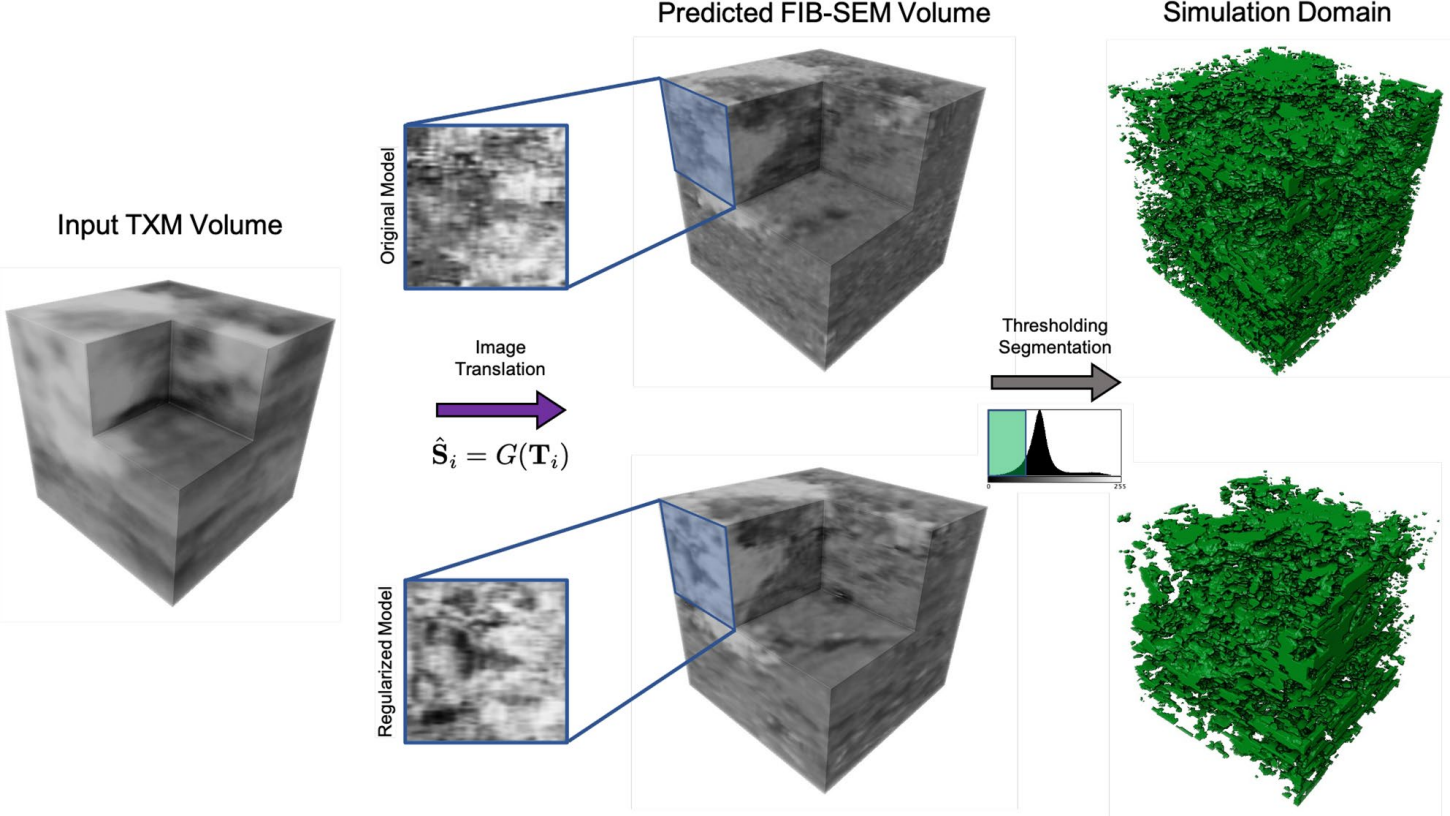
Flow simulation domain generation. A TXM image volume is translated to a FIB-SEM volume using the original (top) and regularized (bottom) SRGAN 4X (Vanilla) model. The image cross-sections show the improved continuity in the generated FIB-SEM volume produced by the regularized model. The image volumes are then segmented into a lower density region using thresholding-based segmentation. This produces a simulation domain that includes kerogen and lower-density minerals where most flow in the volume is assumed to take place. A threshold value is chosen such that the produced lower-density regions form a connected set of voxels. Disconnected voxels are then discarded to create the final flow simulation domain. Software: Powerpoint www.microsoft.com, MATLAB www.mathworks.com, Avizo www.thermofisher.com.

We next synthesize image volumes using the trained models. The volumes are generated by passing each *x*–*y* slice independently through the 2D translation network and stacking along the *z*-axis to form a synthesized volume. Figure 4 shows image volumes generated with the pix2pix (WGAN) and SRGAN 4x (Vanilla) models for the original models, regularized models, and regularized models post-processed with median filtering. As shown in the image volumes, the *z*-regularization improves *z*-direction continuity, and the median filtering reduces “jittering” between image slices in the *z*-direction. We also see that the regularized models contain fewer low density regions, but as shown in Fig. 5, the low density regions are more continuous between slices than for the original models.

We evaluate the quantitative similarity metrics for 3D image volume generation by synthesizing 5 $128^3$ volumes from the test set TXM volume slices and computing the metrics for the *x*–*y* direction (the image generation plane) and the *x*–*z* and *y*–*z* directions (orthogonal to the image generation plane). We compute four image descriptors for each image in the synthesized volumes and test set: the area *A*(*X*), perimeter *P*(*X*), and mean curvature $\chi (X)$ for the low-density regions, and the joint pixel value distribution $p(T_{ij}, S_{ij})$. We then calculate the Kullback-Leibler divergence (KL divergence) between the synthesized and test set image descriptor distributions. The distribution of these descriptors should be similar between the test set images and the synthesized images along all image planes. Therefore, a smaller value of KL divergence indicates better model performance.

Table 2 summarizes the results for the unpaired image prediction metrics. The results for the gray level co-occurrence probability distribution show similar results regardless of the model used. The models with *z*-regularization show a significant improvement in the perimeter and Euler characteristic metrics. However, the area metric showed worse performance for the regularized models, likely because regularized models produce fewer low-density regions. Results also show that post-processing with a median filter produces worse results for the pix2pix (WGAN) model but improves the perimeter and Euler characteristic results for the SRGAN 4x (Vanilla) model.

**Table 2:** Image similarity metrics for volume generation.

Model	*A*(*X*)		*P*(*X*)		$\chi (X)$		$p(T_{ij}, S_{ij})$
	*x*/*y*	*x*/*z*, *y*/*z*	*x*/*y*	*x*/*z*, *y*/*z*	*x*/*y*	*x*/*z*, *y*/*z*	
**Baseline models**							
No Model	3.82	7.67	8.47	8.42	5.37	**1**.**31**	8.44
Feedforward CNN	1.93	**5**.**54**	**1**.**36**	19.17	3.80	23.05	7.98
pix2pix (Vanilla)	4.32	7.59	2.07	8.15	2.34	23.04	8.06
pix2pix (WGAN)	6.42	9.55	7.60	2.87	3.16	22.31	8.20
SRCNN 4x	4.33	9.03	5.12	7.84	2.00	21.75	8.07
SRCNN 2x	2.80	12.46	6.28	22.47	5.08	22.82	9.00
SRGAN 4x (Vanilla)	6.85	9.48	2.63	3.91	3.30	22.76	8.36
SRGAN 4x (WGAN)	7.68	9.69	5.47	1.44	**1**.**73**	11.46	9.03
SRGAN 2x (Vanilla)	**1**.**41**	7.40	2.27	8.58	1.80	22.72	**7**.**95**
SRGAN 2x (WGAN)	6.83	12.98	9.64	2.35	4.59	13.58	8.29
*z*-**regularization**							
pix2pix (WGAN)	7.65	14.02	10.33	3.34	4.38	16.67	8.89
SRGAN 4x (Vanilla)	9.08	9.99	3.54	1.13	2.30	8.41	8.56
*z* **-regularization + post-processing**							
pix2pix (WGAN)	9.29	16.02	12.78	4.28	4.04	7.24	–
SRGAN 4x (Vanilla)	10.88	12.86	5.05	**0**.**72**	1.94	5.32	–

Metric represents the KL divergence between the distribution of image features computed for the test set image patches and slices from each image plane for synthesized volumes. Lesser is better for all metrics shown. Statistics are computed over 5 total $128^3$ volumes. For the area and perimeter, the *z* regularization and post-processing decrease performance for the *x*–*y* plane and produce similar results for the orthogonal *x*–*z* and *y*–*z* planes. Both *z*-regularization and post-processing show a significant improvement for the Euler characteristic results.

### Flow in reconstructed volumes

**Figure 6:**
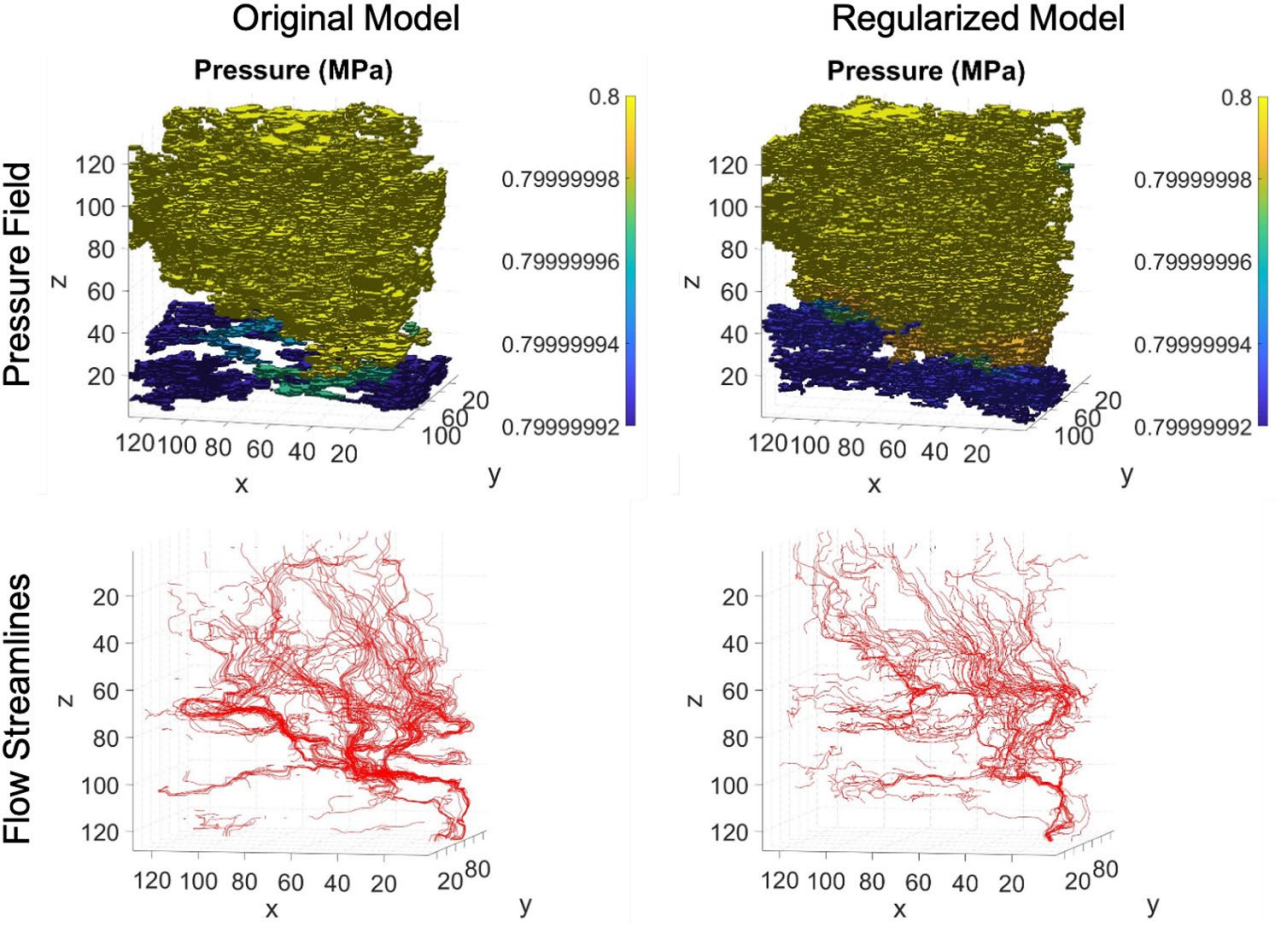
Comparison of pressure fields and flow streamlines in the original volume and in post-processed volumes created with a regularized model. The different models produce substantially different results in flow paths and pressure fields. The original model has more active cells in the simulation domain and therefore produces more flow streamlines. The streamlines for the regularized model, however, have greater continuity in the *z* direction. Software: Powerpoint www.microsoft.com, MATLAB www.mathworks.com.

To demonstrate the application of the presented volume generation approach for evaluating petrophysical properties from nondestructive image data, we simulate flow of methane through volumes generated using the SRGAN 4x (Vanilla) model with and without regularization. This workflow is shown in Fig. 5. Using a three-dimensional, nineteen-velocity (D3Q19) lattice Boltzmann method (LBM), described in the Supplementary Information (SI), we simulate flow of methane in the *z*-direction at a temperature of 370 K, inlet pressure of 0.8 MPa inlet pressure, a pressure drop of $0.8\times 10^{-7}$ MPa, and a kinematic viscosity of $10^{-4}\; \text {cm}^2/\text {s}$. The flow simulations use a second-order slip boundary condition^45,46^. This boundary treatment captures slip velocities in complex pore geometries accurately via the inclusion of local Knudsen numbers^47^. Results from the pressure computations are shown in Table 3 and visualizations of the pressure field and streamlines are shown in Fig. 6. We observe that the choice of model may substantially affect the morphology of the low density regions and therefore the Knudsen number and apparent permeability. The apparent permeability values for both models are reasonable for a shale sample at the rock fabric scale, showing that the proposed image processing method enables both flow visualization and accurate computation of flow properties from non-destructive image data.

**Table 3:** Computed flow results.

Model	Kn	*k* (d)	$\phi$	$\phi _{\text {connected}}$
SRGAN 4x – Original	0.100	$4.10 \times 10^{-7}$	20.7%	18.7%
SRGAN 4x – Regularized	0.0770	$8.14\times 10^{-8}$	18.9%	17.4%

Flow through the generated domains is simulated using LBM simulation. Results show permeability values on the order of ${\mathscr {O}}(10^{1} \text { nd})$ to ${\mathscr {O}}(10^{2} \text { nd})$, which is the expected order of magnitude for apparent permeability for a shale volume at the rock fabric scale. The permeability computations show that the proposed image translation approach is capable of producing realistic simulation domains for evaluating petrophysical properties from nondestructive image data.

## Discussion

Both super-resolution and image translation models are shown to predict FIB-SEM image patches effectively from TXM input images. We also observed that for the GAN models, using the WGAN loss function tended to improve performance in terms of PSNR, SSIM, and STSIM, at the expense of synthesizing fewer low-density regions and reduced performance for low-density region segmentation. This presents a significant challenge when analyzing or visualizing flow through shale volumes, because most of the flow is assumed to take place in the kerogen and low-density mineral regions.

The *z*-regularization approach introduced is promising for improving 3D reconstruction. The image prediction results show that the *z*-regularization term has a minimal effect on the 2D quantitative error metrics while improving performance for some 3D image similarity metrics. In particular, *z*-regularization improves the Euler characteristic results that correlate with flow properties in the porous medium. Therefore this regularization is promising for replicating 3D structures in flow simulation domains. While *z*-regularization is shown to carry many advantages for 3D volume reconstruction, there remains room for improving particularly the quantitative performance of these models and applying this approach in related domains such as medical imaging. An additional significant drawback is that these models take much longer to train due to the extra backpropagation steps required to compute the Jacobian-vector products used in the regularization term.

The simulation results demonstrate the practical application of our models to visualize and characterize flow in a shale rock volume using only non-destructive imaging data. The choice of model, however, may impact the computed apparent permeability and flow paths in a given volume. LBM simulations predict slightly smaller apparent permeability and Knudsen numbers for the regularized model, likely due to the complex pore space connectivity in the computational domain. Removal of active cells and creating disconnected flow paths reduces the ease of flow through the domain, which manifests in simulations as a reduced overall velocity and a less permeable medium.

We assume here that the active cells do not contain organic matter, e.g. kerogen, and their entire volume is available to flow. Although the computed apparent permeabilities are reasonable for a shale sample at the rock fabric-scale, the results may represent an upper bound as a result of this assumption. In future work, we will incorporate the effects of organic content and low permeability regions in the simulation models. The approach presented here could be used to reconstruct low-density regions at the ${\mathscr {O}}(10^1 \text { nm})$ scale, then a generated pore structure obtained from high-resolution images (e.g. transmission electron microscopy images^15^) super-imposed on the low-density regions to obtain a simulation domain at the ${\mathscr {O}}(10^{0}\text { nm})$ scale. Similar approaches have been applied to other multiscale source rocks^48^, and presents a promising future direction for nondestructive image-based characterization of geological porous media.

## Methods

### Multimodal shale image dataset

The dataset consists of paired TXM and FIB-SEM images and is described in detail elsewhere^19^. The TXM images were acquired at beamline 6-2c of the SLAC National Accelerator Laboratory (SLAC) using X-rays at 8 keV. The TXM projections were reconstructed with TXM-Wizard^49^ to produce an image volume of isotropic resolution of 31.2 nm/px. The FIB-SEM images were acquired on a FEI XL 835 DualBeam FIB/SEM instrument that milled and collected cross-sectional images perpendicular to the main axis of the cylinder. Instrument energy was 4kV, and the resolution was 33.6 nm/px in the *x*–*y* plane and variable *z* direction from 30 up to 50 nm. The TXM images were rescaled to be identical to FIB-SEM pixel resolution and aligned to achieve a paired image dataset. The final dataset contains 149 aligned 2D TXM and FIB-SEM grayscale image slices and a 3D TXM image volume consisting of 419 image slices. In both modalities, darker areas denote low density material or features that include: fractures, pores, organic matter and kerogen. Lighter areas denote high density minerals such as carbonates, silicates, barite, and pyrite.

### Image prediction models

We use image translation^37–39^ and SISR^33,34^ models to predict FIB-SEM images from TXM images. These models are divided into two main families referred to as feedforward CNNs and CGANs. Feedforward CNNs map the input image $I^{in}$ to the output image $I^{out}$ using a CNN $G(I^{in}) = {\hat{I}}^{out}$. These models are trained according to the objective:

$$\begin{aligned} G^* = \arg \min _G \sum _{i} || G(T_i) - S_i||_1 \end{aligned}$$

where $T_i$ is the input TXM image and $S_i$ is the ground truth FIB-SEM image. We train using $L_1$ loss because this has been shown to produce sharper images than $L_2$ loss^38^. This model is effectively a nonlinear regression model where the CNN models the mapping between the input data and response values. The architecture for the neural network $G(\cdot )$ can take many forms. In the feedforward CNN model, we use a 9-block ResNet architecture^37^. In the SRCNN model, the $T_i$ image is downsampled by 2x or 4x and $G(\cdot )$ is the SR-ResNet architecture^34^.

We also use the pix2pix^38,39^ and SRGAN^34^ CGAN models. These models consist of a generator network $G(\cdot )$ that predicts the FIB-SEM image and a discriminator network $D(\cdot )$ that evaluates the quality of an output image or pair of input/output images as being real or synthetic. These models are trained according to the objective as

$$\begin{aligned} G^* = \arg \; \min _G \; \max _D \quad \frac{1}{m} \sum _{i=1}^m \underbrace{{\mathscr {L}}_{GAN}(G, D)}_{\text {Style/texture}} + \lambda _{Sim} \underbrace{|| G(T_i) - S_i ||_1}_{\text {Image similarity}} \end{aligned}$$

We experiment with the vanilla GAN loss^40^ as

$$\begin{aligned} {\mathscr {L}}_{GAN}(G,D) = \log D(T_i, S_i) + \log (1 - D(T_i, G(T_i))) \end{aligned}$$

and the Wasserstein GAN (WGAN) loss with gradient penalty^50,51^ as

$$\begin{aligned} {\mathscr {L}}_{GAN}(G,D) = D(T_i, G(T_i)) - D(T_i, S_i) + \lambda _{GP} (||\nabla D(T_i, t G(T_i) + (1-t)S_i)||_2 - 1)^2 \end{aligned}$$

All models are implemented in Pytorch^52^, trained using a framework based on code for the pix2pix and CycleGAN^38,39^ models, optimized using the Adam optimizer^53^, and trained for 75 epochs total with a batch size of 10,000 randomly sampled $128 \times 128$ image patches. We split the dataset of 149 paired image slices as 101 training, 12 validation, and 36 test slices. The large test set was chosen to ensure that the 36 test set 2D slices spanned 128$^3$ voxel subvolumes of the 3D TXM volume. Image patches for training and testing are chosen to contain at least 75% non-zero pixels and 95% non-artifact pixels. We use a learning rate of $2 \times 10^{-4}$ for the first 50 epochs and anneal the learning rate by factor of 10 for the remaining epochs, as this was found to produce stable training and to train all models to convergence. $\lambda _{GP} = 10$ is used for all WGAN models per results from previous work on WGANs^51^.

### *z*-regularization approach

We propose to reconstruct 3D image volumes with 2D-to-2D image models by enforcing continuity between input image slices, and thereby improve continuity in the *z*-direction of the predicted image volume. Our approach draws inspiration from existing work on robust learning^54^. Robust learning seeks to reduce the sensitivity of the class logits to the input data of the network; in our approach, we seek to reduce the sensitivity of the generator network $G(\cdot )$ to perturbations in the input image.

Our approach assumes that $\nabla _{z} S$ is sparse for any SEM image *S*. Therefore, we enforce continuity in the predicted image volumes by regularizing with $||\nabla _{z} S||_1$. This term, however, is not easily computed. For ${\hat{S}} = G(T)$, where ${\hat{S}}$ is the predicted SEM image and *T* is the input nano-CT image, we observe that

$$\begin{aligned} ||\nabla _{z} {\hat{S}}||_1 \le C \left| \left| \frac{\partial {\hat{S}}}{\partial T } \right| \right| _F^2 \equiv {\mathscr {L}}_{\text {Reg}}(G) \end{aligned}$$

The squared Frobenius norm of the Jacobian can be computed efficiently. During training, we add this term to the original objective function to train a regularized model as

$$\begin{aligned} G^* = \arg \; \min _{G}\; \max _{D} \quad {\mathscr {L}}_{\text {Original}}(G, D) + \lambda {\mathscr {L}}_{Reg}(T, G(T)) \end{aligned}$$

### Paired image similarity metrics

We evaluate paired image similarity using four similarity metrics: peak signal to noise ratio (PSNR), structural similarity index metric (SSIM), structural texture similarity index metric (STSIM) and a segmentation-based similarity metric.

**PSNR:** measures the similarity between images in decibels (dB) and is proportional to the inverse of mean-squared error (MSE). For images with pixel values normalized to have $I_{ij} \in [0,1]$, PSNR is computed as

$$\begin{aligned} \text {PSNR}(I^{\text {gt}}, {\hat{I}}) = 10 \log \frac{1}{MSE(I^{\text {gt}}, {\hat{I}})} \end{aligned}$$

**SSIM:** measures the *structural* similarity between images. SSIM is based on the similarity of spatial statistics of the image and is calculated as

$$\begin{aligned} l(X,Y)&= \frac{2 \mu _X\mu _Y + C_1}{\mu _x^2 + \mu _y^2 + C_1} \\ c(X,Y)&= \frac{ 2\sigma _X \sigma _Y + C_2}{\sigma _X^2 + \sigma _Y^2 + C_2} \\ s(X,Y)&= \frac{\sigma _{XY} + C_3}{\sigma _X \sigma _Y + C_3}\\ SSIM(I^{\text {gt}}, {\hat{I}})&= l(I^{\text {gt}}, {\hat{I}})^\alpha c(I^{\text {gt}}, {\hat{I}})^\beta s(I^{\text {gt}}, {\hat{I}})^\gamma \end{aligned}$$

$C_1$, $C_2$, and $C_3$ are small smoothing factors, $\mu _X$ and $\sigma _X$ are respectively the mean and standard deviation of the pixel values in image patch *X*, and usually $\alpha =\beta =\gamma =1$.

**STSIM:** measures *textural* similarity between images and is based in part on SSIM^55^. STSIM is designed to measure perceptual similarity between images and is computed as

$$\begin{aligned} \rho (X; k,\ell )&= \frac{{\mathbf {E}}[(X_{i,j} - \mu _X)(X_{i+k,j+\ell } - \mu _X)]}{\sigma _X^2} \\ r(X,Y;m,n)&= 1 - 0.5|\rho (X; m,n) - \rho _Y(Y; n,m)| \\ STSIM(I^{\text {gt}}, {\hat{I}})&= l(I^{\text {gt}}, {\hat{I}})^{\frac{1}{4}} c(I^{\text {gt}}, {\hat{I}})^{\frac{1}{4}} r(I^{\text {gt}}, {\hat{I}}; 0,1)^{\frac{1}{4}} r(I^{\text {gt}}, {\hat{I}}; 1,0)^{\frac{1}{4}} \end{aligned}$$

where $\rho _X$ computes the correlation coefficient of the image pixels in image *X* with offset *k* and $\ell$ in the *x* and *y* directions, respectively. The additional terms have been shown^55^ to improve image retrieval by measuring perceptual similarities rather than structural.

**Segmentation-based Image Similarity**: similar inputs should produce FIB-SEM images that are segmentable into the same rock phases^19^. Hence, we evaluate the outputs of the GAN model by segmenting the outputs and comparing this to the segmentation of the ground truth image^38^. Let $Seg_k(\cdot )$ be the segmentation classifier for class *k* that maps input image *I* to a binary mask of the pixels selected for class *k*. We compute the image similarity metric as the Dice score of the segmentation as

$$\begin{aligned} \text {Seg. sim.}(I^{\text {gt}}, {\hat{I}}) = \frac{2 |Seg_k(I^{\text {gt}}) \cap Seg_k({\hat{I}})| + \varepsilon }{|Seg_k(I^{\text {gt}})|+ |Seg_k({\hat{I}})| + \varepsilon } \end{aligned}$$

where $\varepsilon$ is a small smoothing factor to account for empty classes. Here we segment the images into five regions: background, low density, medium density, high density, and surface charging artifacts. The classifier is implemented in Ilastik^56^ and uses a random forest classifier to segment images based on pre-computed image features.

### Unpaired image similarity metrics

The lack of ground truth volumetric data precludes evaluation synthetized image volumes using paired image metrics. We propose to evaluate the 3D reconstruction by measuring the similarity between the distribution of structural features for generated and ground truth images. We compute this metric by using a function that maps an image *I* (either single modality or paired multimodal image) to a scalar value, then compute the Kullback-Leibler Divergence (KL divergence) between the distribution for synthetic images $q(f({\hat{I}}))$ and ground truth images $p(f(I^{\text {gt}}))$. KL divergence is computed as

$$\begin{aligned} \text {KL div.} = \sum _{x \in {\mathscr {X}}} p(x) \log \frac{p(x)}{q(x)} \end{aligned}$$

KL divergence takes values on the range $[0, +\infty )$, so a smaller value indicates a closer match between the probability distributions *q*(*x*) and *p*(*x*).

We compute this metric for features in the *x*–*y* plane (image generation plan) and the *x*–*z* and *y*–*z* planes (orthogonal to the image generation plane). This method assumes that structural features in porous media at the nanoscale are approximately isotropic and therefore the distribution of structural features in all image planes should match the distribution from ground truth test set images. We compute this metric for Minkowski functionals and the pixel value joint distribution.

**Minkowski functionals distributions:** Minkowski functionals measure topological features of images and provide structural descriptors of a solid volume and are a common metric for characterizing porous materials^57^. In two dimensions, there are three Minkowski functionals

$$ \begin{aligned}   {\text{Area:}}\qquad A(X) &  = \int_{X} d x \\    {\text{Perimeter:}}\qquad P(X) &  = \frac{1}{2}\int_{{\partial X}} d x \\    {\text{Euler - Poincare }}\;{\text{ characteristic:}}\qquad \chi (X) &  = \frac{1}{{2\pi }}\int_{{\partial X}} \kappa  (x)dx \\  \end{aligned}  $$

where $\kappa (x) = \frac{1}{R(x)}$ is the inverse of the principal radius. We measure structural similarity of the predicted FIB-SEM images by computing the Minkowski functionals^58^ for the low-density regions segmented with the Ilastik classifier used for the paired similarity metrics.

**Pixel value joint distribution:** we form the joint histogram between TXM and FIB-SEM images and normalize to create a joint probability distribution of pixel values $p(T_{ij}, S_{ij})$, then compute the KL divergence between the pixel value distributions for real and synthetic images.

### Computation of petrophysical properties

We estimate petrophysical properties of source rocks by processing predicted FIB-SEM image volumes into simulation domains then applying digital rock physics techniques to simulate flow through the rock volume. The active cells for the simulation domain are the segmented lower density voxels. We identify and remove disconnected active cells to improve the stability and convergence behavior of the numerical simulations. Using results from LBM flow simulations, we calculate apparent gas permeability as^47^

$$\begin{aligned} k_g = \frac{2\mu L\bar{q_o}p_o}{p_i^2-p_o^2}, \end{aligned}$$

where $\mu$ is the gas dynamic viscosity, *L* is the domain length along the main direction of flow, $\bar{q_o}$ is the average fluid velocity at the outlet, and $p_i$ and $p_o$ are the inlet and outlet pressures, respectively. A shortcoming of this model, with respect to the permeability values, is the underlying assumption that the simulation domain is open pore space. In reality, the low-density regions are mixes of kerogen and pore space. Therefore, this approach provides an upper bound on the permeability rather than an exact number. Nevertheless, the models allow us to visualize possible flow paths through the sample volume and evaluate the impact of different volume reconstruction methods on the computed petrophysical properties.

## Supplementary Information

Supplementary Information.
