# Supplementary material for: 2D-to-3D image translation of complex nanoporous volumes using generative networks
Source: Sci Rep. 2021 Oct 21;11:20768. doi: 10.1038/s41598-021-00080-5 (PMC8531351; doi:10.1038/s41598-021-00080-5)
Supplement: Supplementary file 1 — Supplementary Information. [file 41598_2021_80_MOESM1_ESM.pdf]

Supplementary Information: 2D-to-3D Image Translation of Complex Nanoporous Volumes Using Generative Networks

Timothy I. Anderson[1], Bolivia Vega[2], Jesse McKinzie[3], Saman A. Aryana[3], and Anthony R. Kovscek[2,*]

[1]Stanford University, Electrical Engineering, Stanford, CA, 94305, USA
[2]Stanford University, Energy Resources Engineering, Stanford, CA, 94305, USA
[3]University of Wyoming, Chemical Engineering, Laramie, WY, 82071, USA
[*]kovscek@stanford.edu

Derivation of Regularization Term

To derive the Jacobian regularization term, we begin with equivalence of the 1- and 2-norms as

$$\begin{aligned} ||\nabla_z S||_1 &\leq \alpha ||\nabla_z S||_2 \\ &= \alpha \sum_{i,j} \left(\frac{dS_{ij}}{dz} \right)^2 \end{aligned}$$

From the chain rule, it follows

$$\frac{dS_{ij}}{dz} = \sum_{k,\ell} \frac{dS_{ij}}{dT_{k\ell}} \frac{dT_{k\ell}}{dz}$$

and by the Cauchy-Schwarz inequality, it follows

$$\left(\frac{dS_{ij}}{dz} \right)^2 \leq \sum_{k,\ell} \left(\frac{dS_{ij}}{dT_{k\ell}} \right)^2 \sum_{k,\ell} \left(\frac{dT_{k\ell}}{dz} \right)^2$$

Using that the TXM image has bounded z-direction derivatives, we write

$$\left|\frac{dT_{k\ell}}{dz}\right| \leq C_1$$
$$\left(\frac{dS_{ij}}{dz}\right)^2 \leq C_1^2 N^2 \sum_{k,\ell} \left(\frac{dS_{ij}}{dT_{k\ell}}\right)^2$$
$$\sum_{i,j} \left(\frac{dS_{ij}}{dz}\right)^2 \leq C_1^2 N^2 \sum_{i,j} \sum_{k,\ell} \left(\frac{dS_{ij}}{dT_{k\ell}}\right)^2$$
$$= C_1^2 N^2 ||J_T(S)||_F^2$$

Neural Network Architectures

For the feedforward CNN and pix2pix models, we use a 9-block ResNet model as shown in Fig. 1

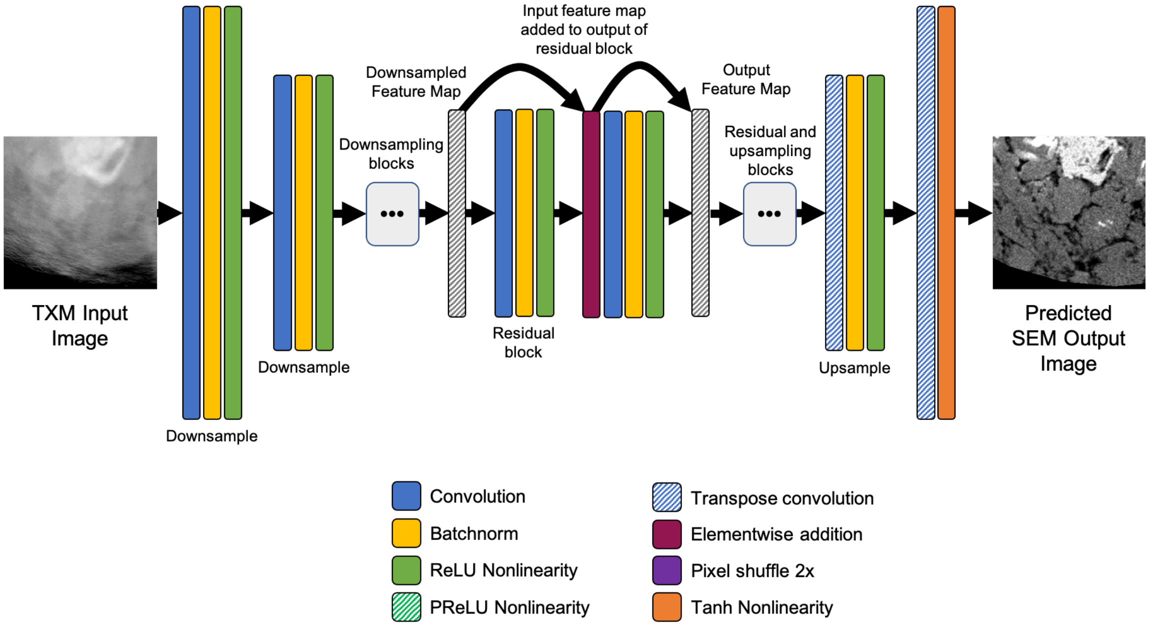

Figure 1: 9-block ResNet architecture.

For the SRCNN and SRGAN models, we use a version of the SR-ResNet model illustrated in Fig. 2.

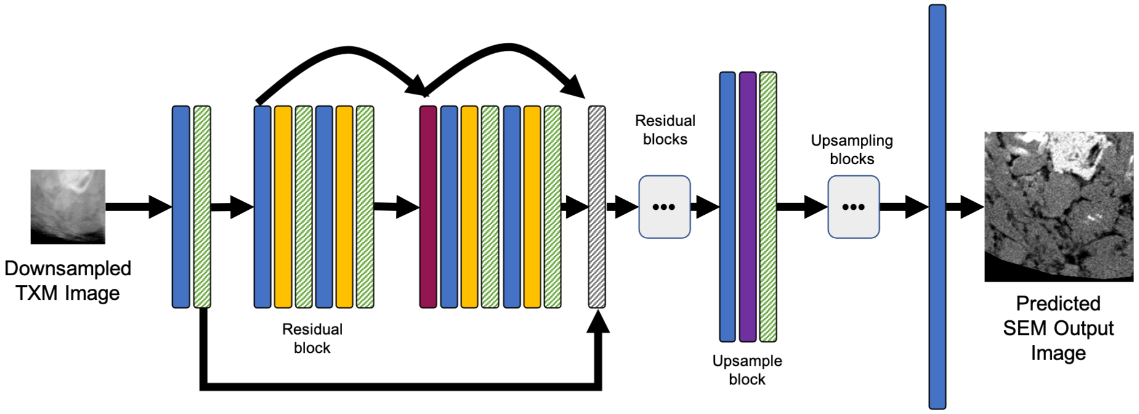

Figure 2: SR-ResNet architecture.

The number of pixel shuffle blocks is determined by the upsampling factor: 2x upsampling requires one pixel shuffle block, 4x upsampling has two pixel shuffle blocks. The SR-ResNet is similar to the 9-block ResNet but with a few notable modifications, particularly the skip connections over the entire chain of residual blocks.

Apparent Permeability via LBM Simulation

This method solves the lattice Boltzmann equation (LBE) given by [1, 2] as

$$f_\alpha(\boldsymbol{x} + c\boldsymbol{e}_\alpha \delta t, t + \delta t) - f_\alpha(\boldsymbol{x}, t) = \Omega_\alpha(f(\boldsymbol{x}, t)), \tag{1}$$

where α represents the index of the discrete velocity and $f_\alpha(\boldsymbol{x}, t)$ is the particle distribution function at position $\boldsymbol{x}$ and time t. For the collision operator, Ω_α, we use the multiple relaxation time (MRT) collision operator [3, 4],

$$\Omega_\alpha = -\sum_\beta (M^{-1} - SM)_{\alpha\beta}(f_\beta - f_\beta^{eq})$$
$$S = \text{diag}(\tau_\rho, \tau_e, \tau_\epsilon, \tau_j, \tau_q, \tau_j, \tau_q, \tau_j, \tau_q, \tau_s, \tau_\pi, \tau_s, \tau_\pi, \tau_s, \tau_s, \tau_s, \tau_m, \tau_m, \tau_m,)^{-1}.$$

where M is a transformation matrix projecting the distribution functions onto a momentum space. S is the collision matrix where τ represents the relaxation time and each subscript denotes to the respective moment. In particular, τ_ρ and τ_j are related to mass and momentum, and τ_e, τ_ϵ, τ_q and τ_s are related to internal energy, internal energy, internal energy square, energy flux and stress tensor. The final two, τ_m and τ_π, are related to the cubic and fourth-order polynomials of the momentum [5]. Specifically, $\tau_p = \tau_j = 1.0$, $\tau_e = 1.19$, $\tau_\epsilon = \tau_\pi = 1.4$, and $\tau_m = 1.98$. τ_q is related to slip velocity and is discussed later in this section and τ_s is related to the fluid viscosity where [6, 7]

$$\tau_s = \frac{1}{2} + \sqrt{\frac{6}{\pi}} \frac{NK_n}{1 + 2K_n}, \tag{2}$$

where K_n is the local Knudsen number and N is the lattice number along the characteristic length, given by $N = H/\delta x$. H is calculated by doubling the distance between a node and the boundary

for nodes on the medial axis and is the distance from the nearest medial axis node for all other nodes. At equilibrium, the particle distribution function is given by [8]

$$f_\alpha^{eq} = w_\alpha \rho \left[1 + \frac{\boldsymbol{e}_\alpha \cdot \boldsymbol{u}}{c_s^2} + \frac{(\boldsymbol{e}_\alpha \cdot \boldsymbol{u})^2}{2c_s^4} - \frac{\boldsymbol{u}^2}{2c_s^2} \right], \tag{3}$$

where $c_s^2 = 1/3$ is the square lattice speed of sound and w_α are the lattice weights given by

$$w_\alpha = \begin{cases} \frac{12}{36}, & \alpha = 0 \\ \frac{2}{36}, & \alpha \in \{1, 2, \ldots, 6\} \\ \frac{1}{36}, & \alpha \in \{7, 8, \ldots, 18\}. \end{cases} \tag{4}$$

The macroscopic fluid properties are

$$\rho = \sum_\alpha f_\alpha(\boldsymbol{x}, t) \tag{5}$$

$$\boldsymbol{u} = \frac{1}{\rho} \sum_\alpha f_\alpha(\boldsymbol{x}, t) \boldsymbol{e}_\alpha, \tag{6}$$

where ρ is density and $\boldsymbol{u}$ is the velocity. In the above-mentioned D3Q19 discretization scheme of LBM, the distribution function is projected onto a space that is spanned by the leading N Hermite orthonormal basis, H^N [9, 10]. In this projection, we keep the second-order terms in the Hermite expansions. As such, f_α^{eq} is properly projected onto H^2. On the other hand, f_α maintains higher order terms and may not be projected onto H^2. The following regularization process is used to remedy this inconsistency [10, 11]. As part of the regularization algorithm, the distribution function is written as

$$f_\alpha = f_\alpha^{eq} + f'_\alpha, \tag{7}$$

where f'_α is the non-equilibrium piece of the distribution. Projection of f'_α onto the second-order Hermite expansion is given by

$$\widetilde{f}_\alpha = w_\alpha \left[\frac{1}{c_s^2} \mathcal{H}^{(2)} \left(\boldsymbol{e}_\alpha / c_s \sum_\beta f'_\beta \boldsymbol{e}_\beta \boldsymbol{e}_\beta \right) \right] \tag{8}$$

$$\mathcal{H}^{(2)}_{\alpha\beta}(e) = \boldsymbol{e}_\alpha \boldsymbol{e}_\beta - \delta_{\alpha\beta}, \tag{9}$$

where $\mathcal{H}^{(2)}$ is the second order Hermite polynomial and $\delta_{\alpha\beta}$ is the Kronecker delta. Replacing the non-equilibrium piece of f_α with its projection onto the second-order Hermite expansion and substituting the result into the discretized Boltzmann equation (Eq. 1) yields

$$f_\alpha(\boldsymbol{x} + c\boldsymbol{e}_\alpha \delta t, t + \delta t) = f_\alpha^{eq} + \widetilde{f}_\alpha - \sum_\beta (M^{-1} - SM)_{\alpha\beta} (f_\beta - f_\beta^{eq}).$$

Inlet and outlet pressures are held constant [12, 13], particularly 0.8 MPa at the inlet and $0.8 \times (1 - 1 \times 10^{-7})$ MPa at the outlet. No flux boundary conditions are applied to the other four faces of the cube by adding a padding of solid boundary to the respective faces. For solid boundaries, we use a three dimensional extension of the boundary treatment similar to [14, 15]

to capture slip velocity because the reference Knudsen numbers of the domains dictate a slip flow regime ($0.01 < K_N < 0.1$). The second order slip boundary condition is given by [16]

$$u_s = C_1 K_n \frac{\partial u}{\partial n}\bigg|_w + C_2 \frac{\partial^2 u}{\partial n^2}\bigg|_w, \tag{10}$$

The two constants, C_1 and C_2, are slip coefficients, n represents the normal vector to the wall, and the subscript w denotes at the wall. To capture the behavior of this condition, a combination of discrete Maxwell diffusion and half-way bounce back is used, given by

$$f_\alpha = r f_\alpha^{DM} + (1-r) f_\alpha^{BB} \tag{11}$$

$$r = \frac{2C_1}{\sqrt{\frac{6}{\pi}} + C_1}, \tag{12}$$

where r is a ratio that signifies the contribution of the discrete Maxwell diffusion, and f^{DM} and f^{BB} are components of the distribution function from Maxwell diffusion and the bounce-back scheme, respectively. The boundary treatments are given by

$$f_\alpha^{BB} = f_\sigma \tag{13}$$

$$f_\alpha^{DM} = K f_\alpha^{eq}, \tag{14}$$

where σ is the vector opposite α and

$$K = \frac{\sum_{\boldsymbol{\xi}'_\alpha \cdot \boldsymbol{n} < 0} |\boldsymbol{\xi}'_\alpha \cdot \boldsymbol{n}|\, f_\alpha}{\sum_{\boldsymbol{\xi}'_\alpha \cdot \boldsymbol{n} > 0} |\boldsymbol{\xi}'_\alpha \cdot \boldsymbol{n}|\, f_\alpha}. \tag{15}$$

In Eq. 15, $\boldsymbol{\xi}'_\alpha = \boldsymbol{e}_\alpha - u_w$, $\boldsymbol{n}$ is the boundary unit normal vector and $\boldsymbol{u}_w$ is the wall velocity. As mentioned previously, the parameter τ_q is related to the slip velocity and is given by

$$\tau_q = \frac{1}{2} + \frac{3 + \pi(2\tau_s - 1)^2 C_2}{8(2\tau_s - 1)}. \tag{16}$$

References

[1] Chen, S. & Doolen, G. Lattice Boltzmann method for fluid flows. *Annual Review of Fluid Mechanics* **30**, 329–364, DOI: https://doi.org/10.1146/annurev.fluid.30.1.329 (1998).

[2] He, X. & Luo, L.-S. Theory of the lattice Boltzmann method: From the Boltzmann equation to the lattice Boltzmann equation. *Physical Review E* **56**, 6811, DOI: 10.1103/PhysRevE.56.6811 (1997).

[3] Lallemand, P. & Luo, L.-S. Theory of the lattice Boltzmann method: Dispersion, dissipation, isotropy, galilean invariance, and stability. *Phys. Rev. E* **61**, 6546–6562, DOI: 10.1103/PhysRevE.61.6546 (2000).

[4] D'Humieres, D. Multiple–relaxation–time lattice Boltzmann models in three dimensions. *Philosophical Transactions of the Royal Society A* **360**, 437–451, DOI: https://doi.org/10.1098/rsta.2001.0955 (2002).

[5] Suga, K. Lattice Boltzmann methods for complex micro-flows: Applicability and limitations for practical applications. *Fluid Dynamics Research* **45**, 034501, DOI: http://dx.doi.org/10.1088/0169-5983/45/3/034501 (2013).

[6] Michalis, V. K., Kalarakis, A. N., Skouras, E. D. & Burganos, V. N. Rarefaction effects on gas viscosity in the Knudsen transition regime. *Microfluidics and Nanofluidics* **9**, 9847–9853, DOI: https://doi.org/10.1007/s10404-010-0606-3 (2010).

[7] Li, Q., He, Y. L., Tang, G. H. & Tao, W. Q. Lattice boltzmann modeling of microchannel flows in the transition flow regime. *Microfluidics and Nanofluidicis* **10**, 607–618, DOI: https://doi.org/10.1007/s10404-010-0693-1 (2011).

[8] Chen, H., Chen, S. & Matthaeus, W. H. Recovery of the navier-stokes equations using a lattice-gas boltzmann method. *Physical Review A* **45**, R5339, DOI: https://doi.org/10.1103/PhysRevA.45.R5339 (1992).

[9] Zhang, R., Shan, X. & Chen, H. Efficient kinetic method for fluid simulation beyond the navier-stokes equation. *Physical Review E* **74**, 046703, DOI: https://doi.org/10.1103/PhysRevE.74.046703 (2006).

[10] Shan, X., Yuan, X. F. & Chen, H. Kinetic theory representation of hydrodynamics: A way beyond the navier–stokes equation. *Journal of Fluid Mechanics* **550**, 413–441, DOI: doi:10.1017/S0022112005008153 (2006).

[11] Latt, J. & Chopard, B. Lattice boltzmann method with regularized pre-collision distribution functions. *Mathematics and Computers in Simulation* **72**, 165–168, DOI: https://doi.org/10.1016/j.matcom.2006.05.017 (2006).

[12] Zou, Q. & He, X. On pressure and velocity boundary conditions for the lattice boltzmann bgk model. *Physics of Fluids* **9**, 1591–1596, DOI: https://doi.org/10.1063/1.869307 (1997).

[13] Hecht, M. & Harting, J. Implementation of on-site velocity boundary conditions for d3q19 lattice boltzmann simulations. *Journal Statistical Mechanics: Theory and Experiment* **2013**, E02001, DOI: 10.1088/1742-5468/2013/02/e02001 (2013).

[14] Wang, Y. & Aryana, S. A. Pore-scale simulation of gas flow in microscopic permeable media with complex geometries. *Journal of Natural Gas Science and Engineering* **81**, 103441, DOI: https://doi.org/10.1016/j.jngse.2020.103441 (2020).

[15] Frouté, L., Wang, Y., McKinzie, J., Aryana, S. A. & Kovscek, A. R. Transport simulations on scanning transmission electron microscope images of nanoporous shale. *Energies* **13**, 6665 (2020).

[16] Wang, Y. & Aryana, S. A. Coupled confined phase behavior and transport of methane in slit nanopores. *Chemical Engineering Journal* **404**, 126502, DOI: https://doi.org/10.1016/j.cej.2020.126502 (2021).
